# Training and validation of a deep learning U-net architecture general model for automated segmentation of inner ear from CT

**DOI:** 10.1186/s41747-024-00508-3

**Published:** 2024-09-12

**Authors:** Jonathan Lim, Aurore Abily, Douraïed Ben Salem, Loïc Gaillandre, Arnaud Attye, Julien Ognard

**Affiliations:** 1 Department of Neuroradiology—Brest University Hospital, Boulevard Tanguy Prigent, 29200 Brest, France; 2 Inserm, UMR 1101 (Laboratoire de Traitement de l’Information Médicale-LaTIM), Université de Bretagne Occidentale, 5 Avenue Foch, 29200 Brest, France; 3 CLIMAL, 26 Rue du Ballon, 59000 Lille, France; 4 GeodAIsics, Biopolis, 38043 Grenoble, France

**Keywords:** Artificial intelligence, Ear (inner), Image processing (computer-assisted), Neural networks (computer), Tomography (x-ray computed)

## Abstract

**Background:**

The intricate three-dimensional anatomy of the inner ear presents significant challenges in diagnostic procedures and critical surgical interventions. Recent advancements in deep learning (DL), particularly convolutional neural networks (CNN), have shown promise for segmenting specific structures in medical imaging. This study aimed to train and externally validate an open-source U-net DL general model for automated segmentation of the inner ear from computed tomography (CT) scans, using quantitative and qualitative assessments.

**Methods:**

In this multicenter study, we retrospectively collected a dataset of 271 CT scans to train an open-source U-net CNN model. An external set of 70 CT scans was used to evaluate the performance of the trained model. The model’s efficacy was quantitatively assessed using the Dice similarity coefficient (DSC) and qualitatively assessed using a 4-level Likert score. For comparative analysis, manual segmentation served as the reference standard, with assessments made on both training and validation datasets, as well as stratified analysis of normal and pathological subgroups.

**Results:**

The optimized model yielded a mean DSC of 0.83 and achieved a Likert score of 1 in 42% of the cases, in conjunction with a significantly reduced processing time. Nevertheless, 27% of the patients received an indeterminate Likert score of 4. Overall, the mean DSCs were notably higher in the validation dataset than in the training dataset.

**Conclusion:**

This study supports the external validation of an open-source U-net model for the automated segmentation of the inner ear from CT scans.

**Relevance statement:**

This study optimized and assessed an open-source general deep learning model for automated segmentation of the inner ear using temporal CT scans, offering perspectives for application in clinical routine. The model weights, study datasets, and baseline model are worldwide accessible.

**Key Points:**

- A general open-source deep learning model was trained for CT automated inner ear segmentation.
- The Dice similarity coefficient was 0.83 and a Likert score of 1 was attributed to 42% of automated segmentations.
- The influence of scanning protocols on the model performances remains to be assessed.

**Graphical Abstract:**

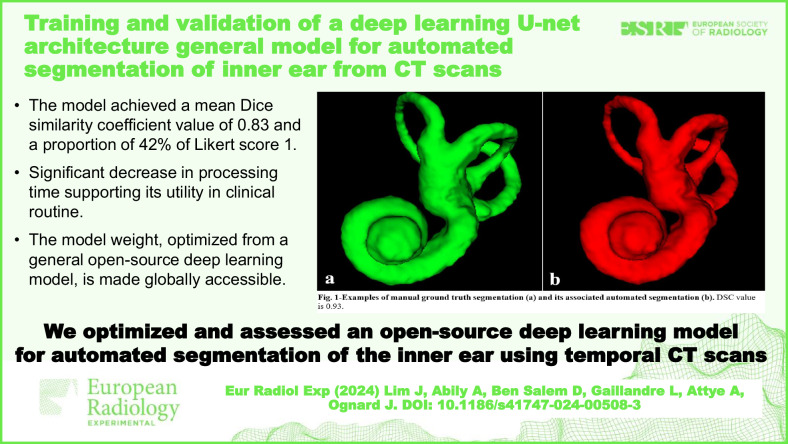

## Background

The inner ear, nestled within the temporal bone, constitutes the innermost part of the ear and includes the vestibule and cochlea, as well as the semicircular canals. Variability in its structure among individuals [1, 2] poses challenges for diagnosis and treatment. Computed tomography (CT) has become crucial for surgeons, providing insights into the spatial relationships between surgical targets and critical structures. Image-guided technologies, such as mastoidectomy [3], cochlear implantation [4–7], and treatment of otosclerosis [8, 9] have proven valuable because of the precision required in these intricate surgeries.

However, effectively visualizing the complex three-dimensional (3D) inner ear anatomy remains difficult [10] on two-dimensional temporal bone CT scans. Manual 3D segmentation of the inner ear is traditionally labor-intensive. Although semi-automatic and automatic methods such as volume rendering [11], growing region, thresholding, edge detection, and model/atlas-based approaches [12] have been explored, human involvement still introduces errors. Although these methods achieve high accuracy, their routine clinical application is limited [3, 4].

Recent advancements in artificial intelligence, particularly deep learning (DL), have notably enhanced 3D inner ear segmentation [13–15]. Convolutional neural networks (CNN), a subset of DL, facilitate novel approaches to automated segmentation in medical imaging by utilizing sophisticated multilayer neural networks [16, 17]. These networks extract complex structural features from input images [18], subsequently generating targeted structures as outputs, showcasing the unique and significant enhancements that DL contributes to computer vision and image segmentation techniques.

This study aimed to assess the development and external validation of a DL general model optimized for automatic inner ear segmentation in external clinical practice. The model quantitative and qualitative evaluations of both healthy and pathological CT scans offer a comprehensive perspective. In this study, weights of the optimized model and study data are made available as open sources.

## Methods

### Ethics

This multicenter study adhered to the ethical guidelines outlined in the Helsinki Declaration and was approved by the National Ethic Committee (Comité d’Ethique pour la Recherche en Imagerie Médicale−CERIM; code: CRM-2310-363; 19/10/2023). All data and patients’ written consents were retrospectively collected. No data were utilized for interventional purposes, or experimentation, or posed any harmful risks to the subjects or study. Our study was classified as non-interventional, observing ethical considerations throughout the research process. A large language model, ChatGPT version 4.0, was used only for translation purposes from native language to English.

### Data collection

This study consisted of two distinct steps. In the initial phase, 146 normal temporal CT scans were randomly chosen from three medical centers for model development and internal validation. These scans, collected between 2016 and 2021 in the same city, aimed to investigate neurological and ear-nose-throat pathologies. Among these, 76 scans were from Center 1 (Morvan Regional University Hospital, Brest, France) using a Somatom Definition A64 scanner (Siemens Healthineers, Erlangen, Germany), 50 from Center 2 (Cavale Blanche General University Hospital, Brest, France) using a Somatom Definition AS + scanner (Siemens Healthineers, Erlangen, Germany), and 20 from Center 3 (Clermont-Tonnerre Military Instruction Hospital, Brest, France) using a Revolution CT scanner (GE Healthcare, Waukesha, United-States). The model was trained and validated on this dataset using a 2:1 ratio, respectively. All scans were obtained from healthy individuals without surgery, malformations, or pathologies.

In the second step, an additional set of 146 CT scans was added to the database at a 1:1 ratio, including healthy and abnormal ears. These scans originated from Center 4 (Grenoble Alpes University Hospital, Grenoble, France) and its affiliated satellite hospitals, collected between 2016 and 2021 from patients with clinical hearing loss and vertigo, and from a variety of scanners. Of the 292 scans in the training dataset, 21 were excluded because of poor spatial resolution or inner ear anatomical issues.

For the external validation test, 70 CT scans were collected from Center 5 (CLIMAL: Medical Imaging Center of Lille Metropolitan Area, Lille, France) in 2022. They were performed on an Aquilion Prime SP scanner (Canon Medical Systems, Otawara, Japan).

Temporal bone protocols from scanners are shown in Supplementary Table 1. All scans were retrospectively collected.

In this study, we employed a bone analysis filter to process each scan with a width of 4,000 HU and a center range of 600 to 800 HU. Image data were provided in DICOM and NifTI-1 formats, employing the “dcm2nii” software (https://www.nitrc.org/projects/dcm2nii/) for conversion purposes.

### Ground truth manual segmentation using ITK-SNAP software

**Fig. 1:**
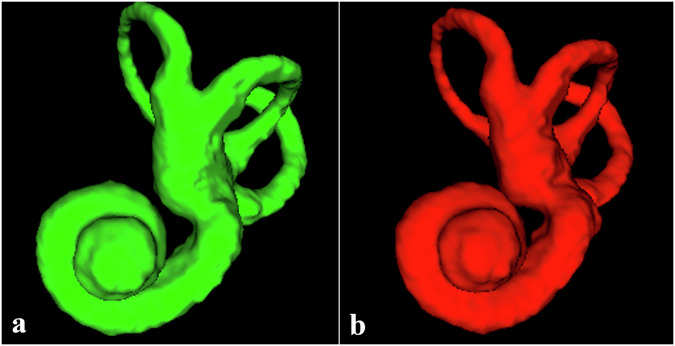
Example of manual ground truth segmentation (**a**) and its associated automated segmentation (**b**). Dice similarity coefficient value = 0.93

ITK-SNAP is a globally available software used for 3D manual segmentation [19] based on edge detection and growing region algorithms. Mask outcomes from manual segmentation were exported as mesh volumes (Fig. 1). The initial set of 146 CT scans was manually segmented by a junior radiologist (A.A.) with four years of experience and reviewed by an expert neuroradiologist (J.O.) with 10 years of experience. Following internal validation, an additional 70 CT scans were manually segmented by another junior radiologist (J.L.) with four years of experience.

### Training and development of a DL model into automated segmentation

#### Automated segmentation workflow and model architecture

The model’s architecture is based on a U-net DL open source [20] and is adaptable to various specific tasks via optimization of the model’s weight. The proposed method utilizes a symmetrical architecture characterized by a sequential arrangement of contraction blocks (encoder), expansion blocks (decoder), and skip connections. Contraction blocks employ convolution layers to reduce the image size while capturing essential features for identification. Conversely, the expansion path consists of a series of up-convolutions and concatenations that merge feature maps to facilitate accurate image segmentation. The inclusion of skip connections between the encoder and decoder enables retrieval of fine details that may be lost during spatial down-sampling. This approach involves the selection and analysis of relevant feature patches within an image for classification, rather than utilizing the entire image. The DL framework is presented in Supplementary material (S1).

#### First training dataset

The first training was performed using 102 randomly selected CT scans from the first set. Ground truth labels were used as the gold standard. All steps were performed on a single dedicated workstation with the following characteristics: GPU 2*GEFORCE GTX1080Ti, Linux-x86_64, NVIDIA Driver Version: 450.119.03, CUDA Version: 11.0; Python 3.8.10; libraries: matplotlib ≥ 3.3.0; scipy ≥ 1.4.0; numpy ≥ 1.18.5; nibabel ≥ 3.1.0. Training hyperparameters are displayed in Supplementary material (S2). It is worldwide available at https://github.com/perslev/.

#### Optimization

A new dataset of 146 CT scans, including abnormal ones, and their additional ground truth labels conducted by senior (R.Q.) and junior (V.D.) radiologists (with 15 and 4 years of experience, respectively) were added to the previous database in a 1:1 ratio, resulting in a total of 292 scans. Of these, 21 scans were excluded from the analysis due to issues such as poor spatial resolution, or absence of anatomical structures of the inner ear. Consequently, an additional training session was conducted using a final comprehensive dataset of 271 CT scans to enhance model performance. The same workstation and hyperparameters, as outlined earlier, were employed during the training process.

**Fig. 2:**
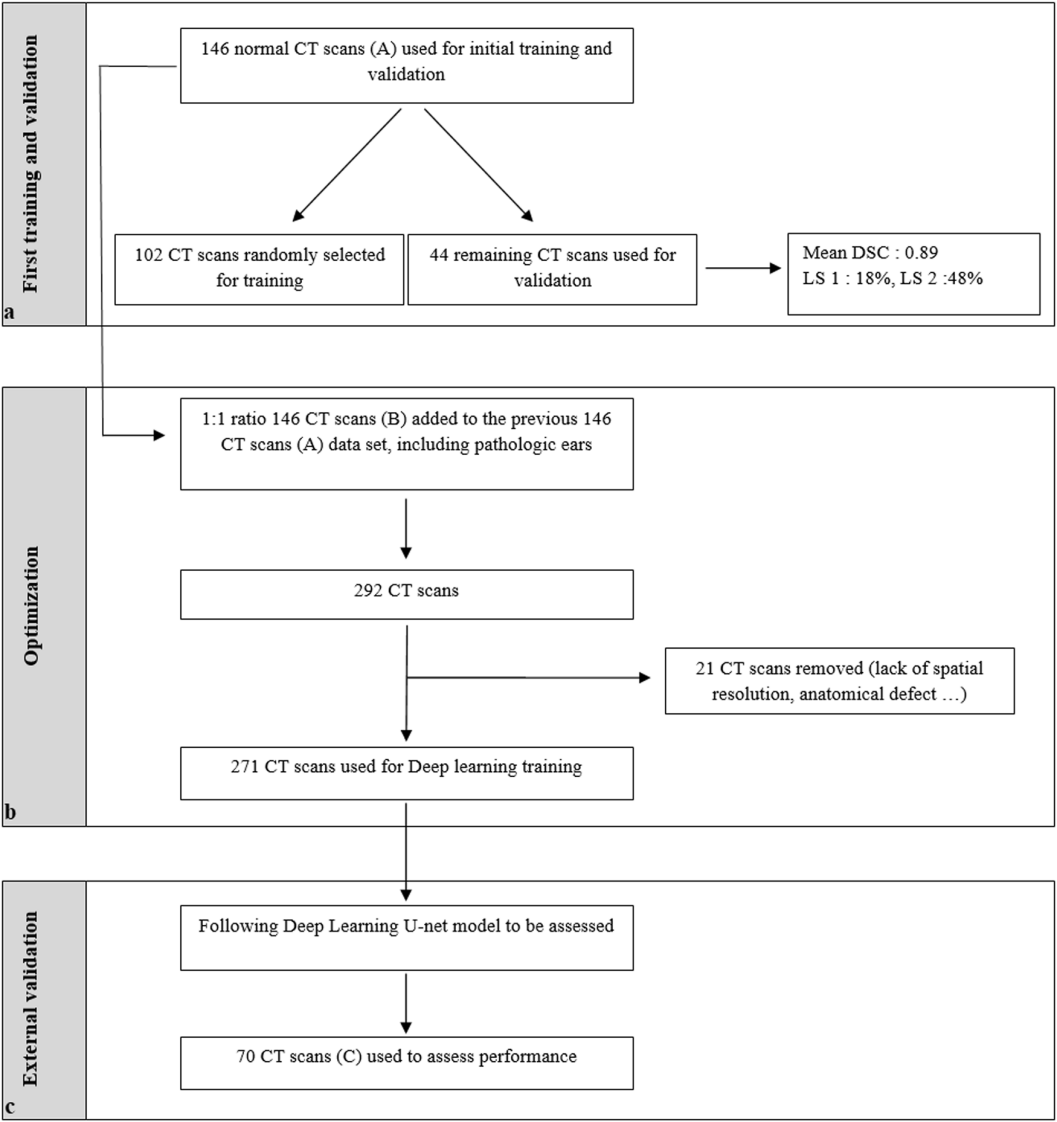
Flowchart of study design associated data management. **a** Centers 1, 2, and 3; **b** Center 4 and satellite hospitals; **c** Center 5, DSC, Dice similarity coefficient; LS, Likert scale

Overall study design associated data management is shown in Fig. 2. The learning of the model over successive iterations follows a logarithmic curve, as demonstrated in Supplementary material (S3).

Finally, a set of 70 healthy and abnormal scans was used as the external validation dataset to assess the performance of the final model.

All automatic segmentations performed for training and validation were post-processed using a sequential approach to remove noise and smooth the volumetric contour. First, a 3D Gaussian filter (sigma: 2.5) was applied, followed by the application of the Otsu thresholding method [21] to the 256-bin filtered image.

The model’s weight, post-comprehensive training, and study datasets are accessible at https://ieee-dataport.org/documents/ct-training-and-validation-series-3d-automated-segmentation-inner-ear-using-u-net. Our study quantitatively evaluated the performance of a DL model by calculating the Dice similarity coefficient (DSC) between manual segmentations and automatic segmentations using the model.

Additionally, qualitative assessment was conducted through a 4-level Likert scale (LS), and we assessed U-net architecture model performance on healthy and abnormal CT scans as a reflection of clinical routine.

### Quantitative assessment using DSC

A quantitative assessment of automatic segmentation was performed using the DSC. DSC is a spatial overlap index that quantifies the concordance between manual ground truth segmentations and automatic segmentations. The DSC ranges from 0 to 1, where a higher DSC value indicates a closer resemblance of the automatic segmentation to the ground truth [22].

### Qualitative assessment using a 4-level LS

**Fig. 3:**
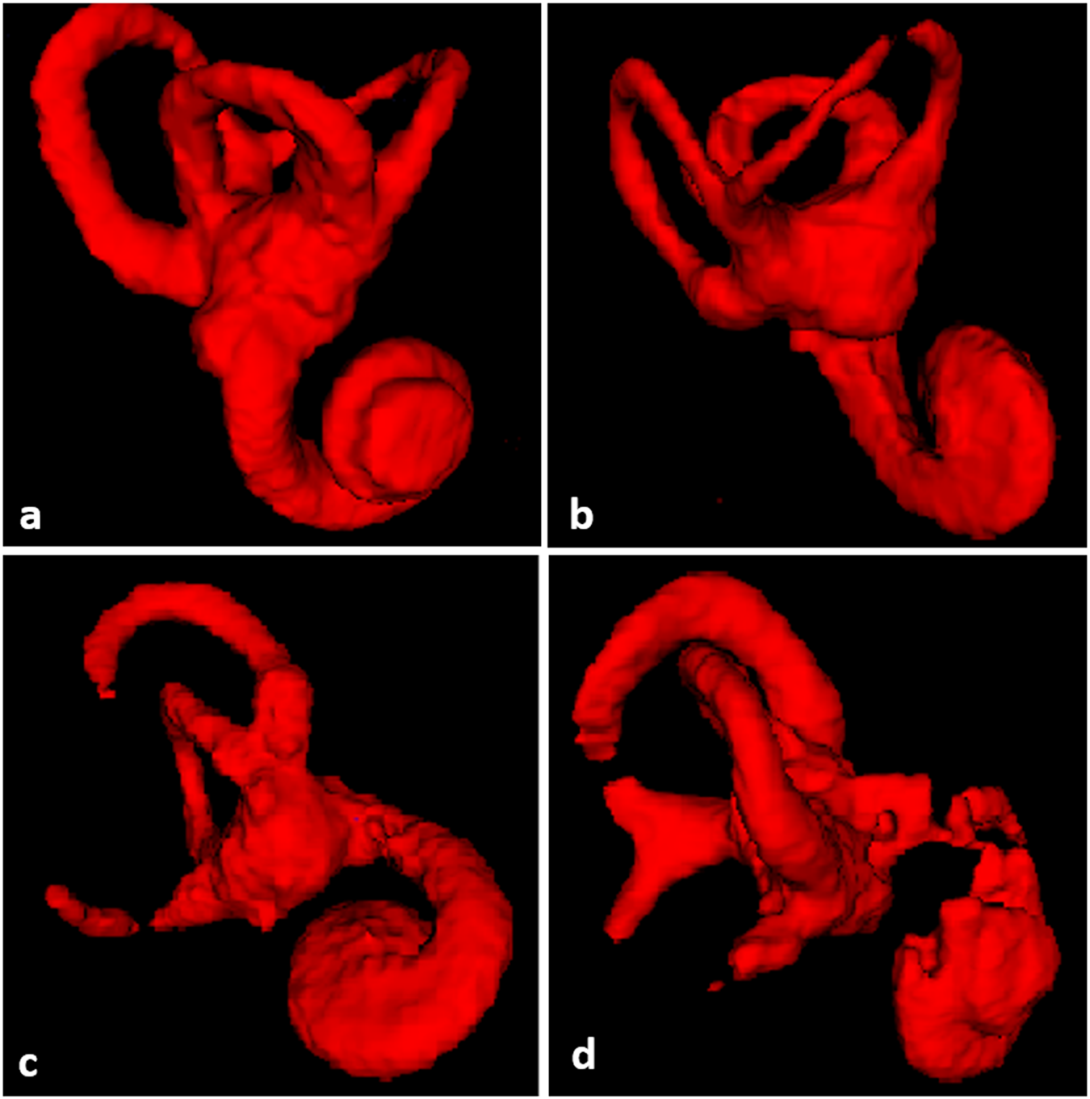
Examples of three-dimensional (3D) volumes with assigned Likert scale score. **a** Score 1: complete 3D volume; **b** Score 2: loss of the upper part of the superior semicircular canal (SSCC); **c** Score 3: loss of the upper part of SSCC and part of the lateral semicircular canal; **d** Score 4: loss of parts of SSCC, lateral semicircular canal and cochlea

A qualitative assessment of the automatic segmentation was conducted using a Likert score. The scale was divided into four levels, based on practical observations of the most observed anomalies in the analyzed 3D volumes, with a score of 1 being the best value and a score of 4 being the worst value (Table 1). The inner ear was divided into five structures: each one of semicircular canal (superior, lateral, and posterior), vestibule, and cochlea. LS 1 was attributed for complete 3D segmentations on mesh volumes. LS 2 was attributed for segmentations missing one structure, LS 3 for segmentations missing two structures, and LS 4 for segmentations missing three structures or presenting substantial inner ear architecture disorganization (Fig. 3). The evaluation was performed by a junior radiologist with four years of experience (J.L.), who was blinded to the DSC values, and conducted on both training and validation datasets.

**Table 1:** Likert scale

Likert Score	Criteria
1	Complete 3D shape: no loss of 3D volume parts
2	Minor loss: at least one structure missing with no substantial architectural disorganization in the inner ear
3	Medium loss: at least two structures missing with no substantial architecture disorganization in the inner ear
4	Major loss: at least three structures missing or any substantial architectural disorganization in the inner ear

Structures of the 3D shape of the inner ear are vestibule, cochlea, and superior, lateral, or posterior semicircular canal

### Statistical analysis

We investigated differences in DSC and LS scores in healthy and abnormal CT scans from training and validation datasets. A Spearman rank-order correlation was conducted to assess the relationship between Likert values and Dice values, for each set.

## Results

### Internal validation set

In the validation phase, the model was run on the remaining 44 CT scans. The average DSC was computed as 0.89, indicating a high level of agreement between the automatic segmentations and the manual ground truth. The LS score of the qualitative evaluation was distributed as follows: LS1 8/44 (18%); LS2 21/44 (48%); LS3 11/44 (25%); and LS4 4/44 (9%). An inversely proportional relationship was observed between the LS score and DSC results.

### Population characteristics

Among the 271 scans in the training set, 171 (63%) represented right ears, 94 (35%) left ears, and 6 (2%) had both sides. After review, CT scans were categorized into three groups: normal (*n* = 192), abnormal (*n* = 79), and incomplete (*n* = 5). The abnormal group included scans with middle ear disorders and inner ear malformations: current or past media otitis (*n* = 25), otosclerosis (*n* = 23) [23], postoperative changes (*n* = 21), and vestibulocochlear dysplasia (*n* = 18), including semicircular canal dehiscence (*n* = 7) or Mondini syndrome (*n* = 3). Incomplete refers to scans missing the upper part of the superior semicircular canal (SSCC) in the acquisition, which could cause segmentation ambiguity.

Within this dataset of 271 scans, 77 (28%) inner ears came from patients who exhibited clinical hearing loss, 92 (34%) had vertigo and 22 (8%) had undergone middle ear surgery.

The validation dataset comprised 70 CT scans. Among these, two (3%) demonstrated SSCC dehiscence, and none were incomplete; 35 (50%) represented right ears, and 35 (50%) represented left ears.

Both training and validation datasets exhibited comparable proportions of normal scans, with 192 (71%) and 55 (79%), respectively, while 79 (29%) of the scans in the training dataset and 15 (21%) in the validation dataset were categorized as abnormal. In-depth analysis of datasets showed similar proportions of inner ear dysplasia, past surgery, and otosclerosis. Subsets were constructed from these categories at risk of segmentation inaccuracies.

Population characteristics are shown in Table 2.

**Table 2:** Number (percentage) according to characteristics of patients in the training and validation datasets

Patients’ characteristics		Training dataset (*n* = 271)	Validation dataset (*n* = 70)
	Left	94 (35%)	35 (50%)
Ear laterality	Right	171 (63%)	35 (50%)
	Both sides	6 (2%)	0 (0%)
Incomplete		5 (2%)	0 (0%)
Abnormal	Inner ear dysplasia	18 (7%)	7 (10%)
	Past surgery	21 (8%)	4 (6%)
	Otosclerosis	23 (8%)	7 (10%)
	Middle ear disorders	79 (29%)	14 (20%)
Normal		192 (71%)	55 (79%)

### Quantitative performance on the validation dataset

The model performance acquired from the validation dataset yielded an average DSC of 0.83 (Table 3), and a DSC median of 0.88, underscoring the prevalence of high DSC values. Subset analysis showed consistency in mean DSC values, as pathological and otosclerosis mean DSC values tend to drop to 0.75 and 0.72 respectively, and the normal subset yielded a higher mean DSC of 0.85. The malformation subset showed the highest mean DSC of 0.90.

**Table 3:** Subgroups analysis and comparison between training and validation datasets

	Training dataset					Validation dataset				
Subsets	*n*	Mean DSC (SD)	Median	Min	Max	*n*	Mean DSC (SD)	Median	Min	Max
Overall dataset	271	0.76 (0.13)	0.79	0.17	0.92	70	0.83 (0.14)	0.88	0.16	0.94
Normal	192	0.77 (0.12)	0.81	0.17	0.92	55	0.85 (0.11)	0.89	0.43	0.94
Pathological	79	0.72 (0.14)	0.76	0.19	0.90	15	0.75 (0.20)	0.85	0.16	0.92
Malformation	18	0.81 (0.12)	0.76	0.48	0.92	7	0.90 (0.04)	0.90	0.87	0.92
Otosclerosis	23	0.73 (0.14)	0.77	0.19	0.90	7	0.72 (0.14)	0.67	0.55	0.89
Incomplete	5	0.63 (0.05)	0.64	0.54	0.66	0	NA	NA	NA	NA

*DSC* Dice similarity coefficient, *NA* Not available, *SD* Standard deviation

The average time required to manually segment each inner ear on a conventional computer was 463 s, resulting in approximately 7 h of segmentation for the validation data. The total training time was 176 h. The average time for automated segmentation of one volume using the GPU was 12 s.

### Qualitative evaluation

**Fig. 4:**
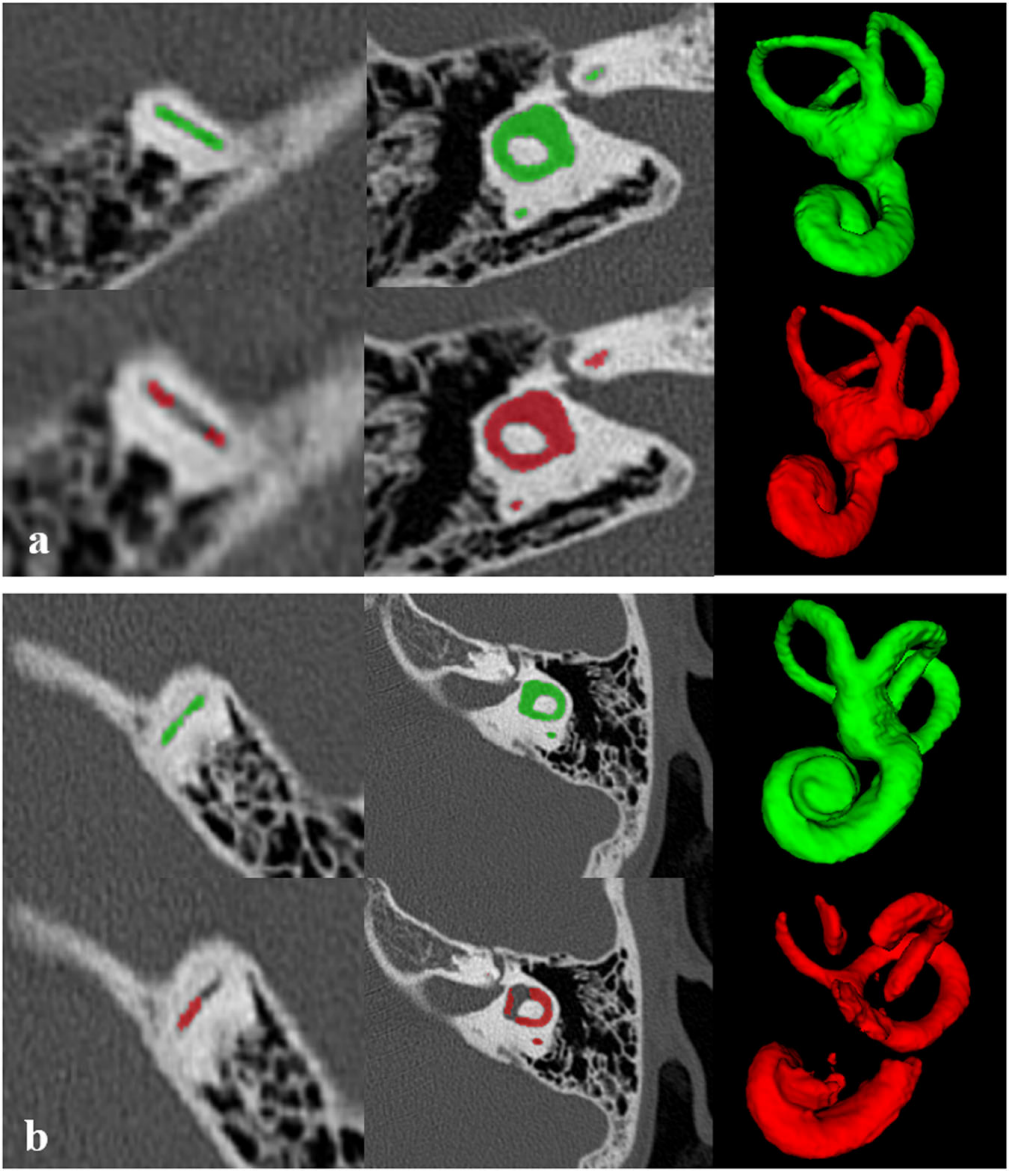
Examples of great and poor predictions. The upper rows show manual segmentation in green. Lower rows show automated segmentation in red. The first and second columns illustrate inner ear parts, while the third column is a three-dimensional rendering. **a** Great prediction: DSC = 0.92; LS score 2 (loss of upper part of SSCC); **b** Poor prediction: DSC = 0.68; LS score 4 (major loss in the cochlea, vestibule, SSCC, and lateral semicircular canal). DSC, Dice similarity coefficient; LS, Likert scale; SSCC, Superior semicircular canal

Among the 70 scans in the validation set, 29 (42%) inner ears were scored 1, indicating a robust level of segmentation accuracy. Notably, 41 (58%) CT scans exhibited segmentation discrepancies in at least one inner ear structure. Within this subset, the majority were assigned LS 4 level, accounting for 19/70 (27%) of the cases. Score 2 was attributed to 17/70 (24%) of the cases, while score 3 was observed in 5/70 (7%) cases. In scans where the sole anomaly was a clinical SSCC dehiscence, without missing concurrent structures, a score of 1 was assigned for matching volume loss. Following clinical analysis of 3D volume rendering, the main information loss (score > 1) was notably concentrated in the semicircular canals, especially the superior canal, contributing to a significant 85% information loss in LS 2, 3, and 4 levels combined. Figure 4 shows examples of structural losses in automated segmentation.

Table 4 summarizes the mean DSC for each LS score. Mean DSC was highest for score 1 and score 2. DSC notably dropped from score 1 to score 4, indicating a marked decline in segmentation accuracy.

**Table 4:** Mean DSC depending on Likert score

Likert score	1 (*n* = 29)	2 (*n* = 17)	3 (*n* = 5)	4 (*n* = 19)
Mean DSC (SD)	0.90 (0.03)	0.90 (0.03)	0.83 (0.06)	0.69 (0.13)

*DSC* Dice similarity coefficient, *SD* Standard deviation

### Performances between training and validation sets

Table 3 shows the DSC subgroups comparison. The overall validation dataset and subsets mean DSCs outperformed those in the training dataset. The overall mean DSC exhibited 0.83 (+ 0.07), the normal subset 0.85 (+ 0.08), the pathological subset 0.75 (+ 0.03), and the malformation subset 0.90 (+ 0.09). However, the otosclerosis subset showed a slight decrease, with a mean DSC of 0.72 (-0.01). These results maintained a consistent standard deviation, except for the malformation subset (0.04 *versus* 0.12). Similarly, the median values aligned with this trend in the overall validation dataset and its subsets, reaching the highest value in the normal subset (0.89). No comparable data were available for the incomplete subset compared to the validation set.

A detailed analysis of the model predictions (Table 5) showed similar proportions of score 1 between the training and validation groups. However, the validation group exhibited more scores of 4. A declining correlation was observed between the mean DSC and LS scores, consistent with the validation group: lower LS levels were associated with higher mean DSC values. The Spearman correlation coefficient was -0.47 for the training set and -0.70 for the validation set (*p* < 0.001 for both).

**Table 5:** Mean DSC correlated to Likert scale scores in training and validation datasets

Likert score		1	2	3	4	Spearman correlation coefficient	*p*-value
Training set (*n* = 271)	*n* (%)	125 (46%)	97 (36%)	39 (14%)	10 (4%)	-0.47	< 0.001
	Mean DSC	0.81	0.73	0.70	0.63		
Validation set (*n* = 70)	*n* (%)	29 (42%)	17 (24%)	5 (7%)	19 (27%)	-0.7	< 0.001
	Mean DSC	0.90	0.90	0.83	0.69		

*DSC* Dice similarity coefficient, *LS* Likert score

## Discussion

### Results analysis

The predictions yielded a high mean DSC of 0.83 compared to the ground truth, with 42% attaining an LS score of 1, showcasing acceptable automatic segmentation under routine clinical conditions. A difference of 0.07 between the mean DSCs of the training (0.76) and validation (0.83) sets highlights robust external validation. The lower DSC values in the training dataset were predominantly due to suboptimal manual segmentation, which adversely affected the quality of the automated segmentations. Nevertheless, after a thorough examination, we did not find cases where inadequate automatic segmentation led to higher DSC values compared to subpar manual segmentation. The unexplained higher prevalence of score 4 in the validation dataset, *i.e*., 19 (27%) *versus* 10 (4%), primarily from normal CT scans, emphasizes the relevance of qualitative evaluation in refining 3D-rendering DL models, pivotal for specialized clinical applications such as image-guided robot surgery and cochlear implants [5, 24, 25]. We believe that, while quantitative metrics assess model precision against the reference standard, qualitative assessments provide insights into the model’s practical clinical applicability, as illustrated through segmentation structural viability. For example, accurately predicting SSCC dehiscence through automatic segmentation is critical and unlikely to impede cochlear image-guided surgery.

### Comparison with literature

Previous studies have demonstrated robust precision, as indicated by the DSC, of automated inner ear CT scan segmentations in clinical practice using mixed pathological and healthy datasets. To our knowledge, prior studies have primarily focused on the quantitative evaluation of DL models, lacking insights into qualitative assessments. Furthermore, limited patient-numbered datasets, specifically in the context of CT scans, constrain insights into broader applicability. The details of these studies are presented in Table 6.

**Table 6:** Comparison of literature

Authors	Our study	Ke et al [28]	Neves et al [26]	Vaidyanathan et al [13]	Hussain et al [16]	Wang et al [27]	Stebani et al [30]	Heutink et al [31]
CNN model architecture	U-net	W-net	AH-net	U-net	U-net	W-net	U-net	U-net
Study modality	Temporal CT	Temporal CT	Temporal CT	MRI	Micro-CT	Temporal CT	Temporal CT	Temporal CT
Pathological data	Yes	No	Yes	Yes	Yes	Yes	No	No
Training dataset (*n*)	271	60	150	944	NA	NA	52	48
Validation dataset (*n*)	70	20	25	99	17	58	47	75
Inner ear mean DSC	0.83	0.91	0.91	0.87	0.90	0.91	0.94	0.90

*CNN* Convolutional neural network, *CT* Computed tomography, *DSC* Dice similarity coefficient, *MRI* Magnetic resonance imaging, *NA* Not available data

Neves et al [26] demonstrated a mean DSC of 0.91 for inner ear 3D rendering using the complex AH-net DL model and noted quicker processing times. The AH-net, more intricate than the U-net due to its attention mechanisms, hybrid architecture, and advanced feature extraction techniques, excels in tasks requiring precision but demands advanced knowledge and specialized resources. In contrast, U-net is simpler and widely supported, particularly for image segmentation [20]. Further recent studies like Wang et al [27] and Ke et al [28] reported a similar DSC using the detailed W-net model, which utilizes dual U-net models to tamper for unsupervised training. Ke et al [28] also included a distinct pediatric CT-scan set and obtained a DSC of 0.91, utilizing data augmentation for the training of the model.

Ding et al [29] proposed to approach bony labyrinth automated segmentation through an image-registration-based pipeline instead of CNN, acquired on cone beam CT (CBCT). An anatomical template was generated based on one out of 16 CBCT images using inverse deformation fields to tamper with the 3D variations of the inner ear. Although it showed a DSC of 0.84 and fast runtime, the absence of extended training and self-taught mistakes like CNNs renders it unable to adjust to anatomical variations. Furthermore, no tests have been reported for operated or pathological ears.

Regarding CNN models, Vaidyanathan et al [13] applied 3D U-Net CNN for inner ear segmentation on MRI scans using high-resolution T2-weighted sequences, showing comparable results to our study with a DSC of 0.87. Their fully trained model in a multicenter study was validated on MRI scans with pathologies or post-therapeutic changes, proving substantial quantitative assessment. Hussain et al [16] employed 17 micro-CT scans from specimen open dataset, achieving a DSC of 0.90, by training their CNN from the ground up, using an auto-context cascaded 2D U-net architecture, allowing for partitioning of input volumes into 2D segmentation architecture, with 3D connected component refinement for segmentation of the inner ear. Additionally, Stebani et al [30] obtained a DSC of 0.94 by developing their U-net CNN on a specimen in-house dataset and subsequently testing it on a set of 10 clinical CT scans. They further evaluated the model performance on additional specimen datasets from open sources to assess its generalizability, which included datasets from CT scans, CBCT, and micro-CT, with achieved DSCs of 0.94, 0.89, and 0.91, respectively. Conversely, Heutink et al [31] used 123 *in vivo* CT scans from a single scanner model for training and validation, but solely focused on cochlea segmentation using a cochlea detection and pixel-wise classification models, performing a DSC of 0.90.

Although our study exhibits the lowest mean DSC value, it is of consideration that all scans used in this study for training and testing were multicenter *in vivo* and in-house CT scans, more widely accessible than micro-CT, and included entire bony labyrinth without requiring additional patches or data augmentation for training supervision, using basic U-net architecture. Moreover, the distinct origin of the training and validation sets underscores the capability for *in vivo* generalization. Also, we reported pathological scans in each dataset. The impact of these on the model performance, especially during training, is still unclear, though a potential decrease in overall performance cannot be disregarded.

### Strengths and limitations of our study

The strength of this multicentric study lies in its incorporation of diverse CT scanners according to manufacturers and models into the model training, enabling the model to train and validate on a broad spectrum of both normal and pathological CT scans from different centers and machine models, underscoring its adaptable and widely applicable nature. This suggests potential utility in clinical settings, reducing variability introduced by manual segmentation and aiding image-guided surgical planning.

Moreover, the model showed promising results for the automatic segmentation of inner ear-challenging lesions such as inner ear dysplasia, highlighting its capability to approach subtle anatomical variations that are clinically significant [32, 33]. The inclusion of more such variations in the model training is likely to enhance its predictive accuracy and clinical relevance, offering perspectives for identifying and classifying minor anatomical differences in future research.

In addition, it stands out for its rigorous qualitative assessment, blinded to DSC values, offering a refined level of evaluation not seen in prior work. While Vaidyanathan et al [13] showed a 67% preference for automatic segmentation in a blind comparison, our use of the LS provided a more nuanced understanding of evaluative subjectivity. Neves et al [26] conducted a similar evaluation but were limited to four CBCTs. Our approach, applied to a broad array of CT scans from both training and validation sets, demonstrated consistent outcomes and negated the likelihood of overfitting. The initial dataset used for internal validation consisted solely of scans from healthy temporal regions, ensuring a consistent baseline for evaluation.

As for limitations, a few should be considered. Although we demonstrated high DSC values from the neural network applied to a complete external CT scans dataset, we could not cover the correlation between technical CT scan parameters (slice thickness, voxel size, pitch, kV, or mAS) with DSC values or the LS score. Given that the validation dataset mostly comprised scans of normal inner ears, it is unlikely to attribute the higher proportion of score 4 in the validation set to confounding pathological voxels exclusively. This observation prompts investigations concerning the influence of scanning protocols on the performance of the model, especially from a qualitative perspective.

In addition, this study exclusively used CT scans, not covering other commonly used CT modalities for temporal bone imaging such as CBCT, particularly in the pediatric population due to concerns about radiation dosage [34]. Recent studies from Ding et al [15] demonstrated consistent accuracy for inner ear automated segmentation using a DL pipeline in CBCT, as well as Benson et al [35] showing higher resolution at a reduced radiation dose in photon-counting detector CT, warranting further exploration in these domains.

Finally, only one junior radiologist manually segmented the validation dataset. Although he was under the supervision of an expert radiologist, the quality of the manual segmentation could not be guaranteed and was likely to influence the performance of the model. Increasing the number of labelers and evaluating their impact on model outcomes are potential avenues for future studies.

### Clinical implications and future strategies

The model yielded substantially reduced processing time (12 s) compared to manual segmentation (463 s), supporting its utility in clinical routine as a radiologist segmentation tool. Furthermore, existing research illustrated the proficiency of DL models in segmenting crucial ear structures [26–29]. Specifically, models have been successfully trained to identify the facial nerve, ossicles, and sigmoid sinus, all of which play indispensable roles in high-precision surgical interventions [9, 24]. Given the demonstrated capabilities, the model presents opportunities for expanding its recognition to additional anatomical features, thus enhancing its clinical utility [30, 36]. Moreover, the emerging use of CT images in augmented reality for otologic surgery [37] is noteworthy, representing a promising avenue for future advancements. While it is still in nascent stages, integration of segmentation DL models into radiological software offers opportunities for advancements in personalized patient care.

On another front, new research has emerged suggesting that the shape of the 3D inner ear may vary depending on sex [38] particularly in individuals below 15 years of age [39]. Bonczarowska et al [39] highlighted sexual dimorphism in the inner ear dimensions, specifically in the width, height, and curvature radius of the cochlea and dimensions of the posterior semicircular canals. As our model’s segmentation closely aligns with ground-truth labels, it is likely that it could be adapted for forensic applications.

While its applicability yields multiple outcomes, it is pivotal to highlight that thorough optimization of the proposed model is required for specific purposes. Also, given its worldwide online availability—through the optimized model weight and base general model—confidence is vested in the feasibility of foreign utilization for inner ear 3D automated segmentation.

## Conclusions

A 3D U-net model architecture was trained and evaluated for the automated and precise segmentation of the inner ear using temporal bone CT scans. The resulting CNN demonstrated high accuracy in segmenting both healthy and pathological CT scans, substantiated through quantitative and qualitative assessments on an external dataset, ensuring its validation and emphasizing qualitative enhancement in developing 3D-rendering DL models. Given its robust performance, we believe this model holds promise for substantial advancements in otology education, surgical simulation, image-guided surgery, and its incorporation into regular clinical practices.

## Supplementary information

**Additional file 1:** **Supplementary Fig. S1.** Deep learning U-net architecture framework. **Supplementary Fig. S2.** Hyperparameters for training session. **Supplementary Fig. S3.** Training and learning of the model following a logarithmic curve. **Supplementary Table 1.** Scanners temporal bone protocols.

## Data Availability

The datasets analyzed during the current study are available in the CT Training and validation series for 3D automated segmentation of the inner ear using U-net architecture deep learning model, https://ieee-dataport.org/documents/ct-training-and-validation-series-3d-automated-segmentation-inner-ear-using-u-net.

## Abbreviations

3D: Three-dimensional
CBCT: Cone beam computed tomography
CNN: Convolutional neural network
CT: Computed tomography
DL: Deep learning
DSC: Dice similarity coefficient
LS: Likert scale
SSCC: Superior semicircular canal

## References

[1] Kontorinis G, Lenarz T (2022) Superior semicircular canal dehiscence: a narrative review. J Laryngol Otol 136:284–292. 10.1017/S0022215121002826 34615564 10.1017/S0022215121002826

[2] Dhanasingh A, Dietz A, Jolly C, Roland P (2019) Human inner-ear malformation types captured in 3D. J Int Adv Otol 15:77–82. 10.5152/iao.2019.6246 31058598 10.5152/iao.2019.6246 PMC6483443

[3] Zagzoog N, Yang VXD (2018) State of robotic mastoidectomy: literature review. World Neurosurg 116:347–351. 10.1016/j.wneu.2018.05.194 29870847 10.1016/j.wneu.2018.05.194

[4] Auinger AB, Dahm V, Liepins R, Riss D, Baumgartner WD, Arnorldner C (2021) Robotic cochlear implant surgery: imaging-based evaluation of feasibility in clinical routine. Front Surg 8:742219. 10.3389/fsurg.2021.742219 34660683 10.3389/fsurg.2021.742219 PMC8511493

[5] Mueller F, Hermann J, Weber S, O’Toole Bom Braga G, Topsakal V (2021) Image-based planning of minimally traumatic inner ear access for robotic cochlear implantation. Front Surg 8:761217. 10.3389/fsurg.2021.761217 34901143 10.3389/fsurg.2021.761217 PMC8655094

[6] Caversaccio M, Wimmer W, Anso J et al (2019) Robotic middle ear access for cochlear implantation: First in man. PLoS One 14:e0220543. 10.1371/journal.pone.0220543 31374092 10.1371/journal.pone.0220543 PMC6677292

[7] Wang J, Liu H, Ke J et al (2020) Image-guided cochlear access by non-invasive registration: a cadaveric feasibility study. Sci Rep 10:18318. 10.1038/s41598-020-75530-7 33110188 10.1038/s41598-020-75530-7 PMC7591497

[8] Nguyen Y, Bernardeschi D, Sterkers O (2018) Potential of robot-based surgery for otosclerosis surgery. Otolaryngol Clin North Am 51:475–485. 10.1016/j.otc.2017.11.016 29502730 10.1016/j.otc.2017.11.016

[9] Parra C, Trunet S, Granger B et al (2017) Imaging criteria to predict surgical difficulties during stapes surgery. Otol Neurotol 38:815–821. 10.1097/MAO.0000000000001417 28414695 10.1097/MAO.0000000000001417

[10] Alenzi S, Dhanasingh A, Alanazi H, Alsanosi A, Hagr A (2021) Diagnostic value of 3D segmentation in understanding the anatomy of human inner ear including malformation types. Ear Nose Throat J 100:675S–683S. 10.1177/0145561320906621 32050777 10.1177/0145561320906621

[11] Xianfen D, Siping C, Changhong L, Yuanmei W (2005) 3D semi-automatic segmentation of the cochlea and inner ear. Conf Proc IEEE Eng Med Biol Soc 2005:6285–6288. 10.1109/IEMBS.2005.1615934 17281704 10.1109/IEMBS.2005.1615934

[12] Kirsch V, Nejatbakhshesfahani F, Ahmadi SA, Dieterich M, Ertl-Wagner B (2019) A probabilistic atlas of the human inner ear’s bony labyrinth enables reliable atlas-based segmentation of the total fluid space. J Neurol 266:52–61. 10.1007/s00415-019-09488-6 31422454 10.1007/s00415-019-09488-6

[13] Vaidyanathan A, van der Lubbe MFJA, Leijenaar RTH et al (2021) Deep learning for the fully automated segmentation of the inner ear on MRI. Sci Rep 11:2885. 10.1038/s41598-021-82289-y 33536451 10.1038/s41598-021-82289-y PMC7858625

[14] Minnema J, van Eijnatten M, Kouw W, Diblen F, Mendrik A, Wolff J (2018) CT image segmentation of bone for medical additive manufacturing using a convolutional neural network. Comput Biol Med 103:130–139. 10.1016/j.compbiomed.2018.10.012 30366309 10.1016/j.compbiomed.2018.10.012

[15] Ding AS, Lu A, Li Z et al (2023) A self-configuring deep learning network for segmentation of temporal bone anatomy in cone-beam CT Imaging. Otolaryngol Head Neck Surg 169:988–998. 10.1002/ohn.317 36883992 10.1002/ohn.317 PMC11060418

[16] Hussain R, Lalande A, Girum KB, Guigou C, Bozorg Grayeli A (2021) Automatic segmentation of inner ear on CT-scan using auto-context convolutional neural network. Sci Rep 11:4406. 10.1038/s41598-021-83955-x 33623074 10.1038/s41598-021-83955-x PMC7902630

[17] Zhang D, Noble JH, Dawant BM (2018) Automatic detection of the inner ears in head CT images using deep convolutional neural networks. Proc SPIE Int Soc Opt Eng 10574:1057427. 10.1117/12.2293383 31007337 10.1117/12.2293383 PMC6474381

[18] Sahiner B, Pezeshk A, Hadjiiski LM et al (2019) Deep learning in medical imaging and radiation therapy. Med Phys 46:e1–e36. 10.1002/mp.13264 30367497 10.1002/mp.13264 PMC9560030

[19] Yushkevich PA, Pashchinskiy A, Oguz I et al (2019) User-guided segmentation of multi-modality medical imaging datasets with ITK-SNAP. Neuroinformatics 17:83–102. 10.1007/s12021-018-9385-x 29946897 10.1007/s12021-018-9385-x PMC6310114

[20] Perslev M, Dam EB, Pai A, Igel C (2019) One network to segment them all: a general, lightweight system for accurate 3D medical image segmentation. In: Shen D et al (eds) Medical Image Computing and Computer Assisted Intervention—MICCAI 2019. Lecture Notes in Computer Science, 11765. Springer, Cham, 30–38 10.1007/978-3-030-32245-8_4

[21] Otsu N (1979) A threshold selection method from gray-level histograms. IEEE Trans Syst Man Cybern 9:62–66. 10.1109/TSMC.1979.4310076 10.1109/TSMC.1979.4310076

[22] Dice LR (1945) Measures of the amount of ecologic association between species. Ecology 26:297–302. 10.2307/1932409 10.2307/1932409

[23] Mangia LRL, Coelho LOM, Carvalho B, de Oliveira AKP, Hamerschmidt R (2021) Imaging studies in otosclerosis: an up-to-date comprehensive review. Int Arch Otorhinolaryngol 25:e318–e327. 10.1055/s-0040-1715149 33968239 10.1055/s-0040-1715149 PMC8096512

[24] Ishiyama A, Risi F, Boyd P (2020) Potential insertion complications with cochlear implant electrodes. Cochlear Implants Int 21:206–219. 10.1080/14670100.2020.1730066 32079506 10.1080/14670100.2020.1730066

[25] Al Saadi M, Heuninck E, De Raeve L, van de Heyning P, Topsakal V (2023) Robotic cochlear implantation in post-meningitis ossified cochlea. Am J Otolaryngol 44:103668. 10.1016/j.amjoto.2022.103668 36323158 10.1016/j.amjoto.2022.103668

[26] Neves CA, Tran ED, Kessler IM, Blevins NH (2021) Fully automated preoperative segmentation of temporal bone structures from clinical CT scans. Sci Rep 11:116. 10.1038/s41598-020-80619-0 33420386 10.1038/s41598-020-80619-0 PMC7794235

[27] Wang J, Lv Y, Wang J et al (2021) Fully automated segmentation in temporal bone CT with neural network: a preliminary assessment study. BMC Med Imaging 21:166. 10.1186/s12880-021-00698-x 34753454 10.1186/s12880-021-00698-x PMC8576911

[28] Ke J, Lv Y, Ma F et al (2023) Deep learning-based approach for the automatic segmentation of adult and pediatric temporal bone computed tomography images. Quant Imaging Med Surg 13:1577–1591. 10.21037/qims-22-658 36915310 10.21037/qims-22-658 PMC10006112

[29] Ding AS, Lu A, Li Z et al (2022) Automated registration-based temporal bone computed tomography segmentation for applications in neurotologic surgery. Otolaryngol Head Neck Surg 167:133–140. 10.1177/01945998211044982 34491849 10.1177/01945998211044982 PMC10072909

[30] Stebani J, Blaimer M, Zabler S, Neun T, Pelt DM, Rak K (2023) Towards fully automated inner ear analysis with deep learning-based joint segmentation and landmark detection framework. Sci Rep 13:19057. 10.1038/s41598-023-45466-9 37925540 10.1038/s41598-023-45466-9 PMC10625555

[31] Heutink F, Koch V, Verbist B et al (2020) Multi-scale deep learning framework for cochlea localization, segmentation and analysis on clinical ultra-high-resolution CT images. Comput Methods Programs Biomed 191:105387. 10.1016/j.cmpb.2020.105387 32109685 10.1016/j.cmpb.2020.105387

[32] Venkatasamy A, Foll DL, Eyermann C et al (2019) Malformations of the lateral semicircular canal correlated with data from the audiogram. Eur Arch Otorhinolaryngol 276:1029–1034. 10.1007/s00405-019-05294-y 30725208 10.1007/s00405-019-05294-y

[33] Zainol Abidin Z, Mohd Zaki F, Kew TY, Goh BS, Abdullah A (2020) Cochlear nerve canal stenosis and associated semicircular canal abnormalities in paediatric sensorineural hearing loss: a single centre study. J Laryngol Otol 134:603–609. 10.1017/S0022215120001334 32713375 10.1017/S0022215120001334

[34] Quintas-Neves M, Saraiva J (2022) Recalling the usefulness of conebeam CT in temporal bone imaging: higher resolution with lower radiation dose. AJNR Am J Neuroradiol 43:E43–E44. 10.3174/ajnr.A7564 36202550 10.3174/ajnr.A7564 PMC9731256

[35] Benson JC, Rajendran K, Lane JI et al (2022) A new frontier in temporal bone imaging: photon-counting detector CT demonstrates superior visualization of critical anatomic structures at reduced radiation dose. AJNR Am J Neuroradiol 43:579–584. 10.3174/ajnr.A7452 35332019 10.3174/ajnr.A7452 PMC8993187

[36] Quatre R, Schmerber S, Attyé A (2024) Improving rehabilitation of deaf patients by advanced imaging before cochlear implantation. J Neurorad 51:145–154. 10.1016/j.neurad.2023.10.002 10.1016/j.neurad.2023.10.002 37806523

[37] Hussain R, Guigou C, Lalande A, Bozorg Grayeli A (2022) Vision-based augmented reality system for middle ear surgery: evaluation in operating room environment. Otol Neurotol 43:385. 10.1097/MAO.0000000000003441 34889824 10.1097/MAO.0000000000003441

[38] Braga J, Samir C, Risser L et al (2019) Cochlear shape reveals that the human organ of hearing is sex-typed from birth. Sci Rep 9:10889. 10.1038/s41598-019-47433-9 31350421 10.1038/s41598-019-47433-9 PMC6659711

[39] Bonczarowska JH, Kranioti EF (2023) Human bony labyrinth as a sex indicator in subadults. Leg Med (Tokyo) 63:102259. 10.1016/j.legalmed.2023.102259 37094514 10.1016/j.legalmed.2023.102259

