# Supplementary material for: Training and validation of a deep learning U-net architecture general model for automated segmentation of inner ear from CT
Source: Eur Radiol Exp. 2024 Sep 12;8:104. doi: 10.1186/s41747-024-00508-3 (PMC11393264; doi:10.1186/s41747-024-00508-3)
Supplement: Supplementary file 1 — Additional file 1: Supplementary Fig. S1. Deep learning U-net architecture framework. Supplementary Fig. S2. Hyperparameters for training session. Supplementary Fig. S3. Training and learning of the model following a logarithmic curve. Supplementary Table 1. Scanners temporal bone protocols. [file 41747_2024_508_MOESM1_ESM.pdf]

Training and Validation of a Deep Learning U-Net Architecture General Model for Automated Segmentation of Inner Ear from CT

ELECTRONIC SUPPLEMENTARY MATERIAL

1. Deep learning U-net architecture framework.

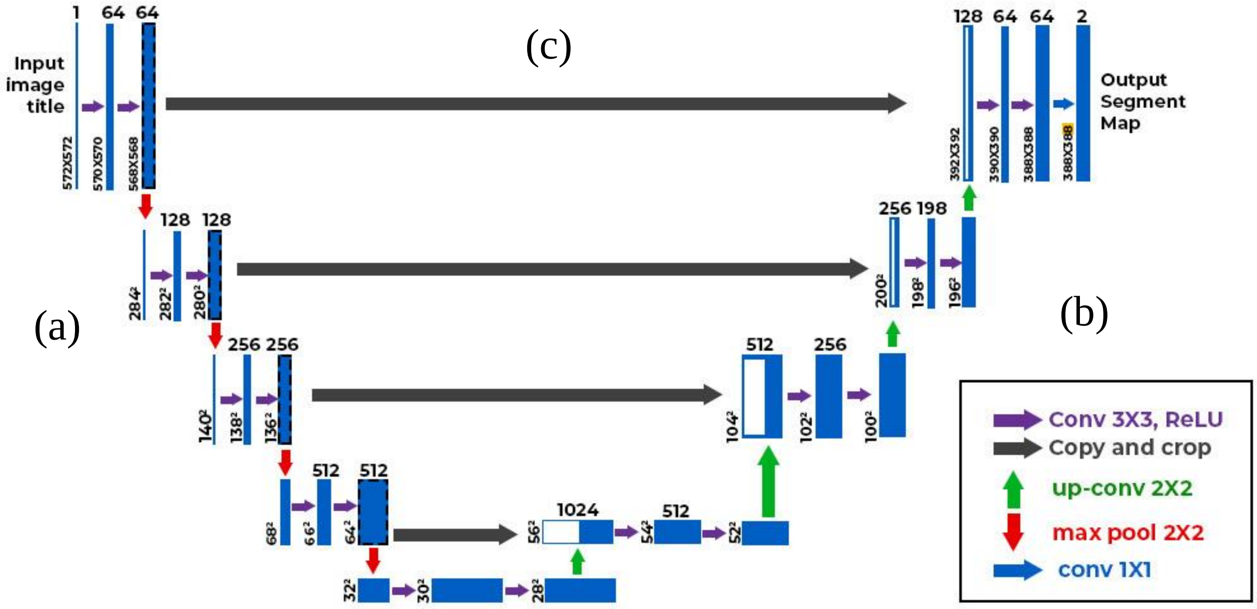

(a) Contraction blocks: Reduce input volume to extract relevant features.
(b) Expansion blocks: Merge feature maps for segmentation accuracy.
(c) Skip connections: Preserve spatial information that might be forfeited during contraction blocks to aid expansion blocks in pinpointing features with enhanced accuracy.

Eur Radiol Exp (2024) Lim J, Abily A, Ben Salem D, Gaillandre L, Attye A, Ognard J.

2. Hyperparameters for training session.

```
__CB_rlop: &RLOP
  # keras.ReduceLROnPlateau
  nickname: "rlop"
  class_name: "ReduceLROnPlateau"
  kwargs: {patience: 2, factor: 0.90, verbose: 1, monitor: "val_dice", mode: "max"}

__CB_tb: &TB
  # tensorboard
  nickname: "tb"
  class_name: "TensorBoard"
  kwargs: {log_dir: './tensorboard', profile_batch: 0}

__CB_mcp_clean: &MCP_CLEAN
  # Model checkpoint
  nickname: "mcp_clean"
  class_name: "ModelCheckPointClean"
  kwargs: {filepath: "./model/@epoch_{epoch:02d}_val_dice_{val_dice:.5f}.h5",
          monitor: "val_dice", save_best_only: true, save_weights_only: true,
          verbose: 1, mode: "max"}

__CB_es: &ES
  # Early stopping
  nickname: "es"
  class_name: "EarlyStopping"

  kwargs: {monitor: 'val_dice', min_delta: 0, patience: 15, verbose: 1 , mode: 'max'}
__CB_timer: &TIMER

  # Train timer callback
  nickname: "timer"
  class_name: "TrainTimer"
  pass_logger: True
  kwargs: {verbose: True}

__CB_csv: &CSV
  # keras.CSVLogger
  nickname: "csv"

  class_name: "CSVLogger"
  kwargs: {filename: "logs/training.csv", separator: ",", append: true}
train_data: &TRAINDATA
  img_subdir: images
  label_subdir: labels
  bg_class: 0

val_data: &VALDATA
  img_subdir: images
  label_subdir: labels
  bg_class: 0
```

```
test_data: &TESTDATA
  img_subdir: images
  label_subdir: labels
  bg_class: 0

aug_data: &AUGDATA
  # Set this to True to include augmented images in the folders described below
  include: False
  img_subdir: images
  label_subdir: labels
  sample_weight: 0.33
  bg_class: 0

build: &BUILD
  #
  # Hyperparameters passed to the Model.build and __init__ methods
  #
  model_class_name: "UNet3D"
  dim: 32
  n_classes: 2
  n_channels: 1
  complexity_factor: 1
  out_activation: "softmax"
  l1_reg: False
  l2_reg: False
  biased_output_layer: True
  depth: 3

fit: &FIT
  #
  # Hyperparameters passed to the Trainer object
  #

  # One of:
  # pre_rotate :
  # live       :
  # iso_live   :
  intrp_style: 'iso_live_3d'
  noise_sd: 0.1

  real_space_span: 50.0
  real_box_dim: 6.25

  # On-the-fly augmentation?
  # Leave empty or delete entirely if not
  augmenters: [
  {cls_name: "Elastic3D",
   kwargs: {alpha: [0, 450], sigma: [12, 25], apply_prob: 0.333}}]

  # Loss function
  loss: "SparseCategoricalCrossentropy"
  metrics: ["sparse_categorical_accuracy"]
```

```
# Pass parameters to the loss function here, e.g. class weights
  # Note: class weights only supported by some loss functions!
  # Leave empty or remove field if loss takes no parameters
  loss_kwargs: {
      # class_weights: [0.2, 0.7, 2.1]
      # gamma: 0.3
  }
  class_weights: False  # Set to True to compute and overwrite class weights in loss_kwargs
automatically

  # Optimization
  batch_size: 8
  n_epochs: 500
  verbose: true
  shuffle_batch_order: true
  optimizer: "Adam"
  optimizer_kwargs: {lr: 5.0e-5, decay: 0.0, beta_1: 0.9, beta_2: 0.999, epsilon: 1.0e-8}

  # Minimum fraction of image slices with FG labels in each mini-batch
  fg_batch_fraction: 0.50
  bg_value: 2pct

  # Normalization, using sklearn.preprocessing scalers
  # NOTE: Applied across full image volumes (after interpolation)
  # Options: MinMaxScaler, StandardScaler, MaxAbsScaler,
  #          RobustScaler, QuantileTransformer, Null
  scaler: "RobustScaler"

  # Callbacks
  callbacks: [*RLOP, *TB, *MCP_CLEAN, *ES, *TIMER, *CSV]
```

3. Training and learning of the model following a logarithmic curve.

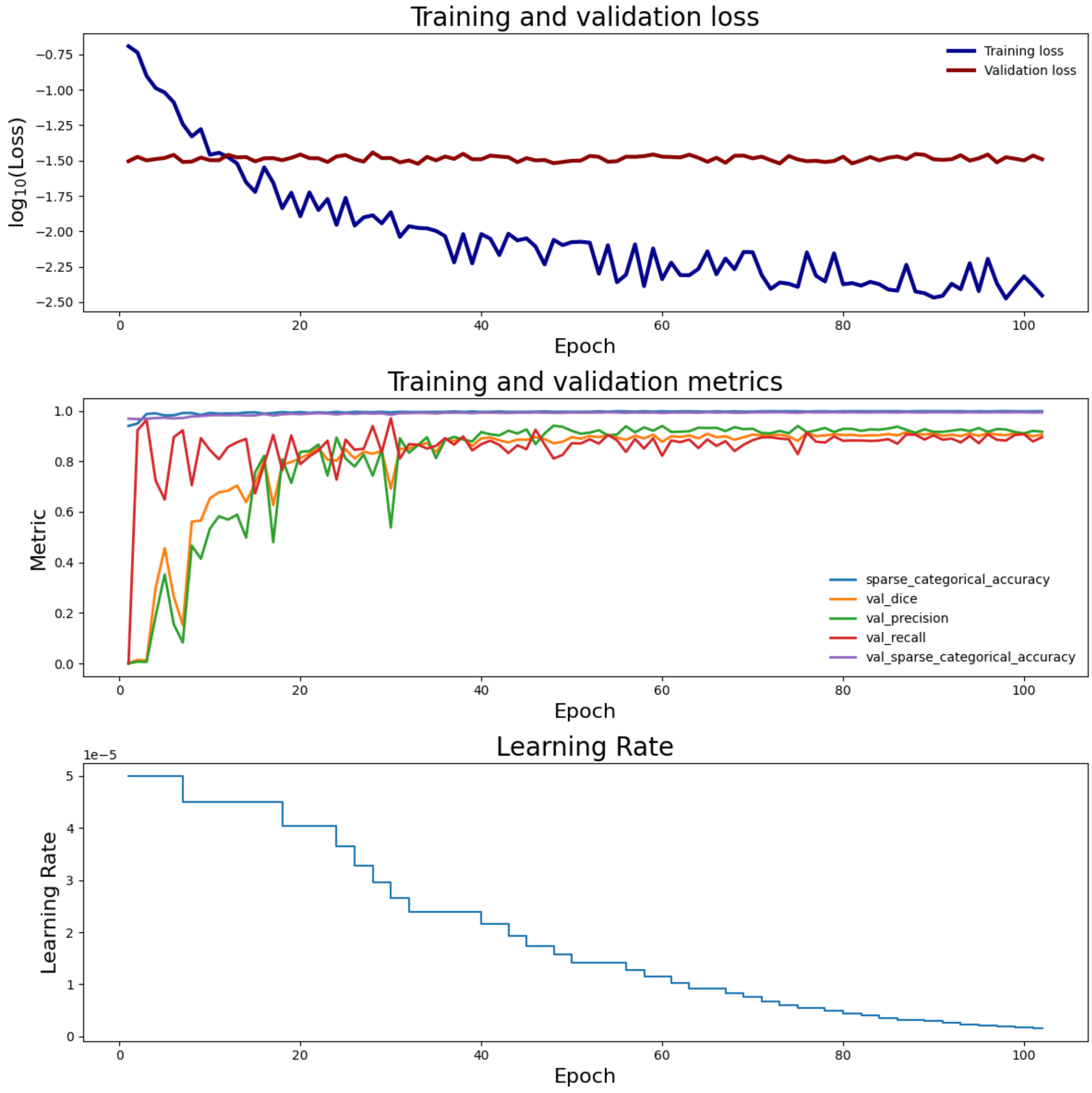

Supplementary Table 1-Scanners temporal bone protocols

	Training dataset							Validation dataset
	SIEMENS Somatom Definition A64	SIEMENS Somatom Definition AS+	GE Revolution	GE Revolution HD	CANON Aquilion Prime SP	PHILIPS Brilliance 40	SIEMENS Somatom Definition Edge	CANON Aquilion Prime SP
Detector (rows)	64	64	64	64	80	64	64	80
Collimation (mm)	0.6×12	0.5×40	0.625×20	0.625×20	40	0.55×2	0.6×12	0.5×40
Tube voltage (kV)	120	135	140	140	120	140	120	120
Tube current (mAs)	320	320	380	380	280	200	320	280
Voxel size (mm)	0.1×0.1×0.3	0.1×0.1×0.3	0.2×0.2×0.3	0.2×0.2×0.3	0.1×0.1×0.2	0.1×0.1×0.3	0.1×0.1×0.3	0.1×0.1×0.2
Pitch factor	0.53	0.63	0.53	0.5	0.7	0.5	0.53	0.7
